# Supplementary material for: A Critical View of the Application of the APEX Software (Aqueous Photochemistry of Environmentally-Occurring Xenobiotics) to Predict Photoreaction Kinetics in Surface Freshwaters
Source: Molecules. 2019 Dec 18;25(1):9. doi: 10.3390/molecules25010009 (PMC7017383; doi:10.3390/molecules25010009)
Supplement: Supplementary file 1 [file molecules-25-00009-s001.zip › 3.2.4_gcc-4.4.0.zip!/3.2.4_gcc-4.4.0/3.2.4_gcc-4.4.0/doc/octave/html/interpreter/index.html]

Untitled


```
START-INFO-DIR-ENTRY
* Octave: (octave).	Interactive language for numerical computations.
END-INFO-DIR-ENTRY
```


Copyright © 1996, 1997, 1999, 2000, 2001, 2002, 2005, 2006,
2007 John W. Eaton.

Permission is granted to make and distribute verbatim copies of
this manual provided the copyright notice and this permission notice
are preserved on all copies.

Permission is granted to copy and distribute modified versions of
this manual under the conditions for verbatim copying, provided that
the entire resulting derived work is distributed under the terms of
a permission notice identical to this one.

Permission is granted to copy and distribute translations of this
manual into another language, under the above conditions for
modified versions.

## Table of Contents

- GNU Octave- Preface
    - Acknowledgements- How You Can Contribute to Octave- Distribution
    - 1 A Brief Introduction to Octave
      - 1.1 Running Octave- 1.2 Simple Examples
          - 1.2.1 Elementary Calculations- 1.2.2 Creating a Matrix- 1.2.3 Matrix Arithmetic- 1.2.4 Solving Systems of Linear Equations- 1.2.5 Integrating Differential Equations- 1.2.6 Producing Graphical Output- 1.2.7 Editing What You Have Typed- 1.2.8 Help and Documentation
          - 1.3 Conventions
            - 1.3.1 Fonts- 1.3.2 Evaluation Notation- 1.3.3 Printing Notation- 1.3.4 Error Messages- 1.3.5 Format of Descriptions
                      - 1.3.5.1 A Sample Function Description- 1.3.5.2 A Sample Command Description- 1.3.5.3 A Sample Variable Description
      - 2 Getting Started
        - 2.1 Invoking Octave from the Command Line
          - 2.1.1 Command Line Options- 2.1.2 Startup Files
          - 2.2 Quitting Octave- 2.3 Commands for Getting Help- 2.4 Command Line Editing
                - 2.4.1 Cursor Motion- 2.4.2 Killing and Yanking- 2.4.3 Commands For Changing Text- 2.4.4 Letting Readline Type For You- 2.4.5 Commands For Manipulating The History- 2.4.6 Customizing `readline`- 2.4.7 Customizing the Prompt- 2.4.8 Diary and Echo Commands
                - 2.5 How Octave Reports Errors- 2.6 Executable Octave Programs- 2.7 Comments in Octave Programs
                      - 2.7.1 Single Line Comments- 2.7.2 Block Comments- 2.7.3 Comments and the Help System
        - 3 Data Types
          - 3.1 Built-in Data Types
            - 3.1.1 Numeric Objects- 3.1.2 Missing Data- 3.1.3 String Objects- 3.1.4 Data Structure Objects- 3.1.5 Cell Array Objects
            - 3.2 User-defined Data Types- 3.3 Object Sizes
          - 4 Numeric Data Types
            - 4.1 Matrices
              - 4.1.1 Empty Matrices
              - 4.2 Ranges- 4.3 Single Precision Data Types- 4.4 Integer Data Types
                    - 4.4.1 Integer Arithmetic
                    - 4.5 Bit Manipulations- 4.6 Logical Values- 4.7 Promotion and Demotion of Data Types- 4.8 Predicates for Numeric Objects
            - 5 Strings
              - 5.1 Escape Sequences in string constants- 5.2 Character Arrays- 5.3 Creating Strings
                    - 5.3.1 Concatenating Strings- 5.3.2 Conversion of Numerical Data to Strings
                    - 5.4 Comparing Strings- 5.5 Manipulating Strings- 5.6 String Conversions- 5.7 Character Class Functions
              - 6 Data Containers
                - 6.1 Data Structures
                  - 6.1.1 Basic Usage and Examples- 6.1.2 Structure Arrays- 6.1.3 Creating Structures- 6.1.4 Manipulating Structures- 6.1.5 Processing Data in Structures
                  - 6.2 Cell Arrays
                    - 6.2.1 Basic Usage of Cell Arrays- 6.2.2 Creating Cell Array- 6.2.3 Indexing Cell Arrays- 6.2.4 Cell Arrays of Strings- 6.2.5 Processing Data in Cell Arrays
                    - 6.3 Comma Separated Lists
                      - 6.3.1 Comma Separated Lists Generated from Cell Arrays- 6.3.2 Comma Separated Lists Generated from Structure Arrays
                - 7 Variables
                  - 7.1 Global Variables- 7.2 Persistent Variables- 7.3 Status of Variables
                  - 8 Expressions
                    - 8.1 Index Expressions- 8.2 Calling Functions
                        - 8.2.1 Call by Value- 8.2.2 Recursion
                        - 8.3 Arithmetic Operators- 8.4 Comparison Operators- 8.5 Boolean Expressions
                              - 8.5.1 Element-by-element Boolean Operators- 8.5.2 Short-circuit Boolean Operators
                              - 8.6 Assignment Expressions- 8.7 Increment Operators- 8.8 Operator Precedence
                    - 9 Evaluation
                      - 9.1 Calling a Function by its Name- 9.2 Evaluation in a Different Context
                      - 10 Statements
                        - 10.1 The `if` Statement- 10.2 The `switch` Statement
                            - 10.2.1 Notes for the C programmer
                            - 10.3 The `while` Statement- 10.4 The `do-until` Statement- 10.5 The `for` Statement
                                  - 10.5.1 Looping Over Structure Elements
                                  - 10.6 The `break` Statement- 10.7 The `continue` Statement- 10.8 The `unwind_protect` Statement- 10.9 The `try` Statement- 10.10 Continuation Lines
                        - 11 Functions and Scripts
                          - 11.1 Defining Functions- 11.2 Multiple Return Values- 11.3 Variable-length Argument Lists- 11.4 Variable-length Return Lists- 11.5 Returning From a Function- 11.6 Default Arguments- 11.7 Function Files
                                        - 11.7.1 Manipulating the load path- 11.7.2 Subfunctions- 11.7.3 Private Functions- 11.7.4 Overloading and Autoloading- 11.7.5 Function Locking- 11.7.6 Function Precedence
                                        - 11.8 Script Files- 11.9 Function Handles, Inline Functions, and Anonymous Functions
                                            - 11.9.1 Function Handles- 11.9.2 Anonymous Functions- 11.9.3 Inline Functions
                                            - 11.10 Commands- 11.11 Organization of Functions Distributed with Octave
                          - 12 Errors and Warnings
                            - 12.1 Handling Errors
                              - 12.1.1 Raising Errors- 12.1.2 Catching Errors
                              - 12.2 Handling Warnings
                                - 12.2.1 Issuing Warnings- 12.2.2 Enabling and Disabling Warnings
                            - 13 Debugging
                              - 13.1 Entering Debug Mode- 13.2 Leavinging Debug Mode- 13.3 Breakpoints- 13.4 Debug Mode- 13.5 Call Stack
                              - 14 Input and Output
                                - 14.1 Basic Input and Output
                                  - 14.1.1 Terminal Output
                                    - 14.1.1.1 Paging Screen Output
                                    - 14.1.2 Terminal Input- 14.1.3 Simple File I/O
                                        - 14.1.3.1 Saving Data on Unexpected Exits
                                        - 14.1.4 Rational Approximations
                                  - 14.2 C-Style I/O Functions
                                    - 14.2.1 Opening and Closing Files- 14.2.2 Simple Output- 14.2.3 Line-Oriented Input- 14.2.4 Formatted Output- 14.2.5 Output Conversion for Matrices- 14.2.6 Output Conversion Syntax- 14.2.7 Table of Output Conversions- 14.2.8 Integer Conversions- 14.2.9 Floating-Point Conversions- 14.2.10 Other Output Conversions- 14.2.11 Formatted Input- 14.2.12 Input Conversion Syntax- 14.2.13 Table of Input Conversions- 14.2.14 Numeric Input Conversions- 14.2.15 String Input Conversions- 14.2.16 Binary I/O- 14.2.17 Temporary Files- 14.2.18 End of File and Errors- 14.2.19 File Positioning
                                - 15 Plotting
                                  - 15.1 Plotting Basics
                                    - 15.1.1 Two-Dimensional Plots
                                      - 15.1.1.1 Two-dimensional Function Plotting
                                      - 15.1.2 Three-Dimensional Plotting
                                        - 15.1.2.1 Three-dimensional Function Plotting- 15.1.2.2 Three-dimensional Geometric Shapes
                                        - 15.1.3 Plot Annotations- 15.1.4 Multiple Plots on One Page- 15.1.5 Multiple Plot Windows- 15.1.6 Printing Plots- 15.1.7 Interacting with plots- 15.1.8 Test Plotting Functions
                                    - 15.2 Advanced Plotting
                                      - 15.2.1 Graphics Objects- 15.2.2 Graphics Object Properties
                                          - 15.2.2.1 Root Figure Properties- 15.2.2.2 Figure Properties- 15.2.2.3 Axes Properties- 15.2.2.4 Line Properties- 15.2.2.5 Text Properties- 15.2.2.6 Image Properties- 15.2.2.7 Patch Properties- 15.2.2.8 Surface Properties- 15.2.2.9 Searching Properties
                                          - 15.2.3 Managing Default Properties- 15.2.4 Colors- 15.2.5 Line Styles- 15.2.6 Marker Styles- 15.2.7 Callbacks- 15.2.8 Object Groups
                                                      - 15.2.8.1 Data sources in object groups- 15.2.8.2 Area series- 15.2.8.3 Bar series- 15.2.8.4 Contour groups- 15.2.8.5 Error bar series- 15.2.8.6 Line series- 15.2.8.7 Quiver group- 15.2.8.8 Scatter group- 15.2.8.9 Stair group- 15.2.8.10 Stem Series- 15.2.8.11 Surface group
                                                      - 15.2.9 Graphics backends
                                                        - 15.2.9.1 Interaction with `gnuplot`
                                  - 16 Matrix Manipulation
                                    - 16.1 Finding Elements and Checking Conditions- 16.2 Rearranging Matrices- 16.3 Applying a Function to an Array- 16.4 Special Utility Matrices- 16.5 Famous Matrices
                                    - 17 Arithmetic
                                      - 17.1 Exponents and Logarithms- 17.2 Complex Arithmetic- 17.3 Trigonometry- 17.4 Sums and Products- 17.5 Utility Functions- 17.6 Special Functions- 17.7 Coordinate Transformations- 17.8 Mathematical Constants
                                      - 18 Linear Algebra
                                        - 18.1 Techniques used for Linear Algebra- 18.2 Basic Matrix Functions- 18.3 Matrix Factorizations- 18.4 Functions of a Matrix- 18.5 Specialized Solvers
                                        - 19 Nonlinear Equations- 20 Diagonal and Permutation Matrices
                                            - 20.1 Creating and Manipulating Diagonal and Permutation Matrices
                                              - 20.1.1 Creating Diagonal Matrices- 20.1.2 Creating Permutation Matrices- 20.1.3 Explicit and Implicit Conversions
                                              - 20.2 Linear Algebra with Diagonal and Permutation Matrices
                                                - 20.2.1 Expressions Involving Diagonal Matrices- 20.2.2 Expressions Involving Permutation Matrices
                                                - 20.3 Functions That Are Aware of These Matrices
                                                  - 20.3.1 Diagonal Matrix Functions- 20.3.2 Permutation Matrix Functions
                                                  - 20.4 Some Examples of Usage- 20.5 The Differences in Treatment of Zero Elements
                                            - 21 Sparse Matrices
                                              - 21.1 The Creation and Manipulation of Sparse Matrices
                                                - 21.1.1 Storage of Sparse Matrices- 21.1.2 Creating Sparse Matrices- 21.1.3 Finding out Information about Sparse Matrices- 21.1.4 Basic Operators and Functions on Sparse Matrices
                                                        - 21.1.4.1 Sparse Functions- 21.1.4.2 The Return Types of Operators and Functions- 21.1.4.3 Mathematical Considerations
                                                - 21.2 Linear Algebra on Sparse Matrices- 21.3 Iterative Techniques applied to sparse matrices- 21.4 Real Life Example of the use of Sparse Matrices
                                              - 22 Numerical Integration
                                                - 22.1 Functions of One Variable- 22.2 Orthogonal Collocation- 22.3 Functions of Multiple Variables
                                                - 23 Differential Equations
                                                  - 23.1 Ordinary Differential Equations- 23.2 Differential-Algebraic Equations
                                                  - 24 Optimization
                                                    - 24.1 Linear Programming- 24.2 Quadratic Programming- 24.3 Nonlinear Programming- 24.4 Linear Least Squares
                                                    - 25 Statistics
                                                      - 25.1 Descriptive Statistics- 25.2 Basic Statistical Functions- 25.3 Statistical Plots- 25.4 Tests- 25.5 Models- 25.6 Distributions- 25.7 Random Number Generation
                                                      - 26 Sets
                                                        - 26.1 Set Operations
                                                        - 27 Polynomial Manipulations
                                                          - 27.1 Evaluating Polynomials- 27.2 Finding Roots- 27.3 Products of Polynomials- 27.4 Derivatives and Integrals- 27.5 Polynomial Interpolation- 27.6 Miscellaneous Functions
                                                          - 28 Interpolation
                                                            - 28.1 One-dimensional Interpolation- 28.2 Multi-dimensional Interpolation
                                                            - 29 Geometry
                                                              - 29.1 Delaunay Triangulation
                                                                - 29.1.1 Plotting the Triangulation- 29.1.2 Identifying points in Triangulation
                                                                - 29.2 Voronoi Diagrams- 29.3 Convex Hull- 29.4 Interpolation on Scattered Data
                                                              - 30 Signal Processing- 31 Image Processing
                                                                  - 31.1 Loading and Saving Images- 31.2 Displaying Images- 31.3 Representing Images- 31.4 Plotting on top of Images- 31.5 Color Conversion
                                                                  - 32 Audio Processing- 33 Object Oriented Programming
                                                                      - 33.1 Creating a Class- 33.2 Manipulating Classes- 33.3 Indexing Objects- 33.4 Overloading Objects
                                                                              - 33.4.1 Function Overloading- 33.4.2 Operator Overloading- 33.4.3 Precedence of Objects
                                                                              - 33.5 Inheritance and Aggregation
                                                                      - 34 System Utilities
                                                                        - 34.1 Timing Utilities- 34.2 Filesystem Utilities- 34.3 File Archiving Utilities- 34.4 Networking Utilities- 34.5 Controlling Subprocesses- 34.6 Process, Group, and User IDs- 34.7 Environment Variables- 34.8 Current Working Directory- 34.9 Password Database Functions- 34.10 Group Database Functions- 34.11 System Information- 34.12 Hashing Functions
                                                                        - 35 Packages
                                                                          - 35.1 Installing and Removing Packages- 35.2 Using Packages- 35.3 Administrating Packages- 35.4 Creating Packages
                                                                                  - 35.4.1 The DESCRIPTION File- 35.4.2 The INDEX file- 35.4.3 PKG\_ADD and PKG\_DEL directives
                                                                          - Appendix A Dynamically Linked Functions
                                                                            - A.1 Oct-Files
                                                                              - A.1.1 Getting Started with Oct-Files- A.1.2 Matrices and Arrays in Oct-Files- A.1.3 Character Strings in Oct-Files- A.1.4 Cell Arrays in Oct-Files- A.1.5 Structures in Oct-Files- A.1.6 Sparse Matrices in Oct-Files
                                                                                          - A.1.6.1 The Differences between the Array and Sparse Classes- A.1.6.2 Creating Sparse Matrices in Oct-Files- A.1.6.3 Using Sparse Matrices in Oct-Files
                                                                                          - A.1.7 Accessing Global Variables in Oct-Files- A.1.8 Calling Octave Functions from Oct-Files- A.1.9 Calling External Code from Oct-Files- A.1.10 Allocating Local Memory in Oct-Files- A.1.11 Input Parameter Checking in Oct-Files- A.1.12 Exception and Error Handling in Oct-Files- A.1.13 Documentation and Test of Oct-Files
                                                                              - A.2 Mex-Files
                                                                                - A.2.1 Getting Started with Mex-Files- A.2.2 Working with Matrices and Arrays in Mex-Files- A.2.3 Character Strings in Mex-Files- A.2.4 Cell Arrays with Mex-Files- A.2.5 Structures with Mex-Files- A.2.6 Sparse Matrices with Mex-Files- A.2.7 Calling Other Functions in Mex-Files
                                                                                - A.3 Standalone Programs
                                                                            - Appendix B Test and Demo Functions
                                                                              - B.1 Test Functions- B.2 Demonstration Functions
                                                                              - Appendix C Tips and Standards
                                                                                - C.1 Writing Clean Octave Programs- C.2 Tips for Making Code Run Faster.- C.3 Tips on Writing Comments- C.4 Conventional Headers for Octave Functions- C.5 Tips for Documentation Strings
                                                                                - Appendix D Contributing Guidelines
                                                                                  - D.1 How to Contribute- D.2 General Guidelines- D.3 Octave Sources (m-files)- D.4 C++ Sources- D.5 Other Sources
                                                                                  - Appendix E Known Causes of Trouble
                                                                                    - E.1 Actual Bugs We Haven't Fixed Yet- E.2 Reporting Bugs- E.3 Have You Found a Bug?- E.4 Where to Report Bugs- E.5 How to Report Bugs- E.6 Sending Patches for Octave- E.7 How To Get Help with Octave
                                                                                    - Appendix F Installing Octave
                                                                                      - F.1 Installation Problems
                                                                                      - Appendix G Emacs Octave Support
                                                                                        - G.1 Installing EOS- G.2 Using Octave Mode- G.3 Running Octave From Within Emacs- G.4 Using the Emacs Info Reader for Octave
                                                                                        - Appendix H GNU GENERAL PUBLIC LICENSE- Concept Index- Function Index- Operator Index

Next: Preface,
Up: (dir)


---

## GNU Octave

This manual documents how to run, install and port GNU Octave, as well
as its new features and incompatibilities, and how to report bugs.
It corresponds to GNU Octave version 3.2.4.

- Preface- Introduction: A brief introduction to Octave.- Getting Started- Data Types- Numeric Data Types- Strings- Data Containers- Variables- Expressions- Evaluation- Statements: Looping and program flow control.- Functions and Scripts- Errors and Warnings- Debugging- Input and Output- Plotting- Matrix Manipulation- Arithmetic- Linear Algebra- Nonlinear Equations- Diagonal and Permutation Matrices- Sparse Matrices- Numerical Integration- Differential Equations- Optimization- Statistics- Sets- Polynomial Manipulations- Interpolation- Geometry- Signal Processing- Image Processing- Audio Processing- Object Oriented Programming- System Utilities- Packages- Dynamically Linked Functions- Test and Demo Functions- Tips and Standards- Contributing Guidelines- Trouble: If you have trouble installing Octave.- Installation: How to configure, compile and install Octave.- Emacs Octave Support
                                                                                      - Copying: The GNU General Public License.- Concept Index: An item for each concept.- Function Index: An item for each documented function.- Operator Index: An item for each documented operator.

--- The Detailed Node Listing ---

Preface

- Acknowledgements- How You Can Contribute to Octave- Distribution

Introduction

- Running Octave- Simple Examples- Conventions

Conventions

- Fonts- Evaluation Notation- Printing Notation- Error Messages- Format of Descriptions

Format of Descriptions

- A Sample Function Description- A Sample Command Description- A Sample Variable Description

Getting Started

- Invoking Octave from the Command Line- Quitting Octave- Getting Help- Command Line Editing- Errors- Executable Octave Programs- Comments

Invoking Octave from the Command Line

- Command Line Options- Startup Files

Command Line Editing

- Cursor Motion- Killing and Yanking- Commands For Text- Commands For Completion- Commands For History- Customizing readline- Customizing the Prompt- Diary and Echo Commands

Comments

- Single Line Comments- Block Comments- Comments and the Help System

Data Types

- Built-in Data Types- User-defined Data Types- Object Sizes

Built-in Data Types

- Numeric Objects- Missing Data- String Objects- Data Structure Objects- Cell Array Objects

Numeric Data Types

- Matrices- Ranges- Single Precision Data Types- Integer Data Types- Bit Manipulations- Logical Values- Promotion and Demotion of Data Types- Predicates for Numeric Objects

Matrices

- Empty Matrices

Integer Data Types

- Integer Arithmetic

Strings

- Escape Sequences in string constants- Character Arrays- Creating Strings- Comparing Strings- Manipulating Strings- String Conversions- Character Class Functions

Creating Strings

- Concatenating Strings- Conversion of Numerical Data to Strings

Data Containers

- Data Structures- Cell Arrays- Comma Separated Lists

Data Structures

- Structure Arrays- Creating Structures- Manipulating Structures- Processing Data in Structures

Cell Arrays

- Creating Cell Arrays- Indexing Cell Arrays- Cell Arrays of Strings- Processing Data in Cell Arrays

Variables

- Global Variables- Persistent Variables- Status of Variables

Expressions

- Index Expressions- Calling Functions- Arithmetic Ops- Comparison Ops- Boolean Expressions- Assignment Ops- Increment Ops- Operator Precedence

Calling Functions

- Call by Value- Recursion

Boolean Expressions

- Element-by-element Boolean Operators- Short-circuit Boolean Operators

Evaluation

- Calling a Function by its Name- Evaluation in a Different Context

Statements

- The `if` Statement- The `switch` Statement- The `while` Statement- The `do-until` Statement- The `for` Statement- The `break` Statement- The `continue` Statement- The `unwind_protect` Statement- The `try` Statement- Continuation Lines

The `switch` Statement

- Notes for the C programmer

The `for` Statement

- Looping Over Structure Elements

Functions and Scripts

- Defining Functions- Multiple Return Values- Variable-length Argument Lists- Variable-length Return Lists- Returning From a Function- Default Arguments- Function Files- Script Files- Function Handles Inline Functions and Anonymous Functions- Commands- Organization of Functions

Function Files

- Manipulating the load path- Subfunctions- Private Functions- Overloading and Autoloading- Function Locking- Function Precedence

Function Handles Inline Functions and Anonymous Functions

- Function Handles- Anonymous Functions- Inline Functions

Errors and Warnings

- Handling Errors- Handling Warnings

Handling Errors

- Raising Errors- Catching Errors

Handling Warnings

- Issuing Warnings- Enabling and Disabling Warnings

Debugging

- Entering Debug Mode- Leaving Debug Mode- Breakpoints- Debug Mode- Call Stack

Input and Output

- Basic Input and Output- C-Style I/O Functions

Basic Input and Output

- Terminal Output- Terminal Input- Simple File I/O- Rational Approximations

Terminal Output

- Paging Screen Output

Simple File I/O

- Saving Data on Unexpected Exits

C-Style I/O Functions

- Opening and Closing Files- Simple Output- Line-Oriented Input- Formatted Output- Output Conversion for Matrices- Output Conversion Syntax- Table of Output Conversions- Integer Conversions- Floating-Point Conversions- Other Output Conversions- Formatted Input- Input Conversion Syntax- Table of Input Conversions- Numeric Input Conversions- String Input Conversions- Binary I/O- Temporary Files- EOF and Errors- File Positioning

Plotting

- Plotting Basics- Advanced Plotting

Plotting Basics

- Two-Dimensional Plots- Three-Dimensional Plotting- Plot Annotations- Multiple Plots on One Page- Multiple Plot Windows- Printing Plots- Interacting with plots- Test Plotting Functions

Two-Dimensional Plots

- Two-dimensional Function Plotting

Three-Dimensional Plotting

- Three-dimensional Function Plotting- Three-dimensional Geometric Shapes

Advanced Plotting

- Graphics Objects- Graphics Object Properties- Managing Default Properties- Colors- Line Styles- Marker Styles- Callbacks- Object Groups- Graphics backends

Graphics Object Properties

- Root Figure Properties- Figure Properties- Axes Properties- Line Properties- Text Properties- Image Properties- Patch Properties- Surface Properties- Searching Properties

Object Groups

- Data sources in object groups- Area series- Bar series- Contour groups- Error bar series- Line series- Quiver group- Scatter group- Stair group- Stem Series- Surface group

Graphics backends

- Interaction with gnuplot

Matrix Manipulation

- Finding Elements and Checking Conditions- Rearranging Matrices- Applying a Function to an Array- Special Utility Matrices- Famous Matrices

Arithmetic

- Exponents and Logarithms- Complex Arithmetic- Trigonometry- Sums and Products- Utility Functions- Special Functions- Coordinate Transformations- Mathematical Constants

Linear Algebra

- Techniques used for Linear Algebra- Basic Matrix Functions- Matrix Factorizations- Functions of a Matrix- Specialized Solvers

Diagonal and Permutation Matrices

- Basic Usage: Creation and Manipulation of Diagonal and Permutation Matrices- Matrix Algebra: Linear Algebra with Diagonal and Permutation Matrices- Function Support: Functions That Are Aware of These Matrices- Example Codes: Some Examples of Usage- Zeros Treatment: The Differences in Treatment of Zero Elements

Basic Usage

- Creating Diagonal Matrices- Creating Permutation Matrices- Explicit and Implicit Conversions

Matrix Algebra

- Expressions Involving Diagonal Matrices- Expressions Involving Permutation Matrices

Function Support

- Diagonal Matrix Functions- Permutation Matrix Functions

Sparse Matrices

- Basics: Creation and Manipulation of Sparse Matrices- Sparse Linear Algebra: Linear Algebra on Sparse Matrices- Iterative Techniques: Iterative Techniques- Real Life Example: Using Sparse Matrices

Basics

- Storage of Sparse Matrices- Creating Sparse Matrices- Information- Operators and Functions

Operators and Functions

- Sparse Functions- Return Types of Operators and Functions- Mathematical Considerations

Numerical Integration

- Functions of One Variable- Functions of Multiple Variables- Orthogonal Collocation

Differential Equations

- Ordinary Differential Equations- Differential-Algebraic Equations

Optimization

- Linear Programming- Quadratic Programming- Nonlinear Programming- Linear Least Squares

Statistics

- Descriptive Statistics- Basic Statistical Functions- Statistical Plots- Tests- Models- Distributions- Random Number Generation

Sets

- Set Operations

Polynomial Manipulations

- Evaluating Polynomials- Finding Roots- Products of Polynomials- Derivatives and Integrals- Polynomial Interpolation- Miscellaneous Functions

Interpolation

- One-dimensional Interpolation- Multi-dimensional Interpolation

Geometry

- Delaunay Triangulation- Voronoi Diagrams- Convex Hull- Interpolation on Scattered Data

Delaunay Triangulation

- Plotting the Triangulation- Identifying points in Triangulation

Image Processing

- Loading and Saving Images- Displaying Images- Representing Images- Plotting on top of Images- Color Conversion

Object Oriented Programming

- Creating a Class- Manipulating Classes- Indexing Objects- Overloading Objects

Overloading Objects

- Function Overloading- Operator Overloading- Precedence of Objects

System Utilities

- Timing Utilities- Filesystem Utilities- File Archiving Utilities- Networking Utilities- Controlling Subprocesses- Process ID Information- Environment Variables- Current Working Directory- Password Database Functions- Group Database Functions- System Information- Hashing Functions

Packages

- Installing and Removing Packages- Using Packages- Administrating Packages- Creating Packages

Creating Packages

- The DESCRIPTION File- The INDEX file- PKG\_ADD and PKG\_DEL directives

Dynamically Linked Functions

- Oct-Files- Mex-Files- Standalone Programs

Oct-Files

- Getting Started with Oct-Files- Matrices and Arrays in Oct-Files- Character Strings in Oct-Files- Cell Arrays in Oct-Files- Structures in Oct-Files- Sparse Matrices in Oct-Files- Accessing Global Variables in Oct-Files- Calling Octave Functions from Oct-Files- Calling External Code from Oct-Files- Allocating Local Memory in Oct-Files- Input Parameter Checking in Oct-Files- Exception and Error Handling in Oct-Files- Documentation and Test of Oct-Files

Sparse Matrices in Oct-Files

- Array and Sparse Differences- Creating Sparse Matrices in Oct-Files- Using Sparse Matrices in Oct-Files

Mex-Files

- Getting Started with Mex-Files- Working with Matrices and Arrays in Mex-Files- Character Strings in Mex-Files- Cell Arrays with Mex-Files- Structures with Mex-Files- Sparse Matrices with Mex-Files- Calling Other Functions in Mex-Files

Test and Demo Functions

- Test Functions- Demonstration Functions

Tips and Standards

- Style Tips: Writing clean and robust programs.- Coding Tips: Making code run faster.- Comment Tips: Conventions for writing comments.- Function Headers: Standard headers for functions.- Documentation Tips: Writing readable documentation strings.

Contributing Guidelines

- How to Contribute- General Guidelines- Octave Sources (m-files)- C++ Sources- Other Sources

Trouble

- Actual Bugs: Bugs we will fix later.- Reporting Bugs- Bug Criteria- Bug Lists- Bug Reporting- Sending Patches- Service

Reporting Bugs

- Bug Criteria- Where: Where to send your bug report.- Reporting: How to report a bug effectively.- Patches: How to send a patch for Octave.

Installation

- Installation Problems

Emacs Octave Support

- Installing EOS- Using Octave Mode- Running Octave From Within Emacs- Using the Emacs Info Reader for Octave
